# Tuning brain networks: The emerging role of transcranial direct current stimulation on structural plasticity

**DOI:** 10.3389/fncel.2022.945777

**Published:** 2022-07-21

**Authors:** Saviana Antonella Barbati, Maria Vittoria Podda, Claudio Grassi

**Affiliations:** ^1^Department of Neuroscience, Università Cattolica del Sacro Cuore, Rome, Italy; ^2^Fondazione Policlinico Universitario A. Gemelli IRCCS, Rome, Italy

**Keywords:** BDNF, brain connectivity, memory, metaplasticity, neurological disorder, stroke, structural plasticity, tDCS

## Abstract

Transcranial direct current stimulation (tDCS) is a non-invasive brain stimulation technique (NIBS) that has been proven to promote beneficial effects in a range of neurological and psychiatric disorders. Unfortunately, although has been widely investigated, the mechanism comprehension around tDCS effects presents still some gaps. Therefore, scientists are still trying to uncover the cellular and molecular mechanisms behind its positive effects to permit a more suitable application. Experimental models have provided converging evidence that tDCS elicits improvements in learning and memory by modulating both excitability and synaptic plasticity in neurons. Recently, among tDCS neurobiological effects, neural synchronization and dendritic structural changes have been reported in physiological and pathological conditions, suggesting possible effects at the neuronal circuit level. In this review, we bring in to focus the emerging effects of tDCS on the structural plasticity changes and neuronal rewiring, with the intent to match these two aspects with the underpinning molecular mechanisms identified so far, providing a new perspective to work on to unveil novel tDCS therapeutic use to treat brain dysfunctions.

## Introduction

Transcranial direct current stimulation (tDCS) is a low-intensity constant electric current given through the scalp by using two electrodes. During stimulation, the current delivered flows through the brain layers and, depending upon the polarity of the applied stimulation, tDCS can have a depolarizing (anodal) or hyperpolarizing (cathodal) effect (Antal et al., 2017). Different electrode configurations are commonly used: (i) unilateral configuration (i.e., one electrode positioned over the target cortical area and the other one over the contralateral supraorbital region or, in some cases, extracephalically); (ii) bilateral (or bihemispheric) configuration (i.e., one electrode positioned over the target cortical area and the other one over the contralateral side).

Early tDCS studies demonstrated how tDCS was able to induce long-lasting and polarity-specific excitability changes in the human motor cortex and changes in synaptic efficacy including long-term potentiation (LTP)-like and long-term depression (LTD)-like effects (Nitsche and Paulus, 2000; unilateral tDCS, 0.286 A/m^2^, 5 min; Lang et al., 2004; unilateral tDCS, 0.286 A/m^2^, 10 min). At the functional level, tDCS application over the motor cortex of healthy subjects enhanced motor learning and motor task performance (Nitsche et al., 2003; unilateral tDCS, 0.286 A/m^2^, 15 min). Later, the application of tDCS over different human brain areas showed multiple beneficial effects on both cognitive and motor function domains primarily modulating short- and long-lasting synaptic plasticity (Di Pino et al., 2014).

Many works and clinical trials provided solid evidence for its use in the neurological disorders, namely, stroke and epilepsy, movement disorders, Parkinson’s disease (PD), and Alzheimer’s disease (AD) (Flöel, 2014). It has also been shown that in aged subjects, anodal tDCS improved memory to a level equal to younger controls (Meinzer et al., 2013; unilateral anodal tDCS 0.286 A/m^2^, 20 min). At last, in the patients with stroke, tDCS was able to ameliorate fine motor control as well as the recovery of upper limb function (Tedesco Triccas et al., 2016). Despite the numerous existing studies, there is still great variability in the protocols adopted as well as in the outcome obtained which makes optimal clinical translation for tDCS difficult. Nevertheless, consistency seems to emerge in cellular/molecular mechanisms engaged by tDCS, especially anodal tDCS.

Indeed, cellular and molecular studies mainly performed in animal models, provided evidence of plasticity mechanisms based on tDCS after-effects thus justifying and supporting its therapeutic potential for brain disorders based on impaired synaptic plasticity (Korai et al., 2021).

Within this frame, in this review, we will summarize recent findings reporting plastic and metaplastic effects of tDCS and the beneath molecular mechanisms, focusing on those involved in the synaptic and dendritic spine changes, which are at the basis of neuronal network and connectivity rearrangements. In particular, we narrowed our review on preclinical and clinical works reporting positive functional outcomes specifically linked to tDCS-induced changes in plasticity and/or connectivity. Unless otherwise stated, the efficacy of treatment has been reported by the cited studies compared with the control groups receiving sham stimulation.

Experimental and clinical results will be discussed and paralleled to pin down a clearer picture of the mechanisms underlying tDCS modulatory effects providing elements to perfection the therapeutic application of this non-invasive brain stimulation technique (NIBS) and to use its properties to shape the plastic elements of the brain.

## Plastic and metaplastic effects of transcranial direct current stimulation

Many works have documented plasticity-related effects of tDCS in animal models. The ability of tDCS to modulate Hebbian plasticity might well explain its positive effects on motor learning and cognitive enhancement observed in animal and human models both in physiological and pathological conditions.

**FIGURE 1:**
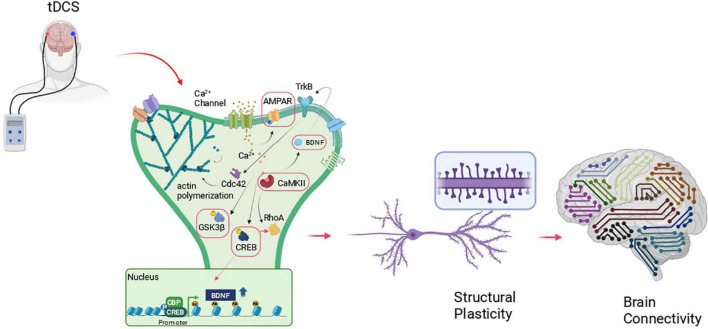
Schematic representation of the molecular cascade at the basis of structural plasticity and its possible recruitment by tDCS. The red boxes indicate those molecules engaged by transcranial direct current stimulation (tDCS)- in different brain areas, in animal models that, so far, have been correlated to changes at synapses (i.e., enhanced LTP, synaptic transmission, dendritic spine density), increased learning and memory and enhanced connectivity. Created with BioRender.com.

Podda et al. (2016) showed increased LTP in the hippocampus of mice subjected to unilateral anodal tDCS (current density, 56 A/m^2^ for 20 min) and shed light on the molecular mechanisms underlying tDCS plastic effects. Basically, tDCS transiently increases intracellular Ca^2+^ initiating a molecular cascade that leads to an increased level of pCREB^*Ser*133^ and its binding on *BDNF* promoter I. This event facilitates CREB binding protein (CBP) recruitment on the promoter region which, in turn, increases H3K9 promoter acetylation thus enhancing *BDNF* exon I expression (Figure 1). Histone acetylation on *BDNF* promoter I was previously shown to affect LTP and long-term memories (Alarcón et al., 2004) and, in keeping with this, Podda and collaborators demonstrated in this study that tDCS improved hippocampus-dependent spatial and recognition memory performance as revealed by the Morris Water Maze and Novel Object Recognition tasks. Consistent with epigenetic regulation, both LTP and memory enhancement induced by anodal tDCS persisted 1 week after the end of the stimulation protocol (Podda et al., 2016).

Hence, while the immediate effects of tDCS can be explained by membrane potential changes, long-term after-effects are the result of intracellular calcium dynamics and secondary synaptic plasticity key elements modulation (Korai et al., 2021). This activity-dependent modulation called “metaplasticity” is fundamental for the modulation and the maintenance of synaptic strength that is at the basis of the learning process (Cooper and Bear, 2012). Interestingly, unilateral anodal tDCS of the hippocampus has been shown to induce BDNF-mediated priming after-effects on synaptic plasticity and memory, making synapses susceptible to LTP induction in the rodent hippocampus (Podda et al., 2016; Yu et al., 2019; 0.1 A/m^2^, 30 min).

tDCS metaplastic outcomes have been observed in several clinical studies. Indeed, unilateral anodal tDCS (0.43 A/m^2^, 15 min) over the primary motor cortex (M1) followed by a repetitive transcranial magnetic stimulation (TMS) protocol elicits polarity-dependent facilitation on motor-evoked potentials (MEPs) in the healthy human subjects (Cosentino et al., 2012). Similarly, Bocci et al. (2014) showed that unilateral anodal tDCS (0.43 A/m^2^, 15 min) preconditioning of the primary visual cortex followed by the application of TMS repetitive stimulation protocol can induce and modulate synaptic strength in healthy subjects, proving the occurrence of tDCS metaplastic mechanisms (Hurley and Machado, 2017). At last, Monte-Silva et al. (2013) showed the presence of metaplastic interactions in response to two consecutive unilateral sessions of anodal tDCS of M1 (0.57 A/m^2^, 13 min) when these were separated by a time interval resulting in an increase in MEPs as a metaplastic outcome in healthy subjects.

In line with this, Barbati et al. (2020) showed that unilateral anodal tDCS of the mouse M1 (35.4 A/m^2^ for 20 min, single daily sessions for 3 consecutive days) induced metaplastic-like effects resulting in motor skill performance improvements similarly to what observed in humans (Reis et al., 2008; Roji et al., 2015; Angius et al., 2018). Specifically, the authors demonstrated that tDCS-induced increase in LTP at layer II-III synapses and such effect was accompanied by increased phosphorylation at Ser831 on the subunit 1 of AMPAR (pGluA1^*Ser*831^), a residue particularly important for the channel permeabilization and LTP expression providing the evidence of tDCS effect on LTP through a metaplastic modification.

## Transcranial direct current stimulation modulation of structural plasticity

Physiologically dendritic spines respond to synaptic plasticity stimulation by modifying their structural and functional features, contributing to learning and memory formation (Rogerson et al., 2014). On the contrary, synaptic dysfunction has been widely recognized as a prodromal sign of both neurodegenerative (Taoufik et al., 2018) and neuropsychiatric disorders (Wang et al., 2018). Indeed, the perturbation of synaptic structure and function is thought to be the basis of the clinical symptoms and the progressive appearance of cognitive deficits (Spires-Jones and Knafo, 2012).

Spines undergo activity-dependent changes playing a fundamental role in LTP and LTD through spine enlargement and shrinkage, respectively (Harris et al., 2003). In addition to this, synaptic activity not only regulates the number of dendritic spines but also their shape and volume by modifying their internal sub-structures (Bosch and Hayashi, 2012; Alimohamadi et al., 2021). Indeed, the modulation local actin polymerization together with its interaction with scaffolding molecules, modifies spine architecture by sturdly positioning channels, cell adhesion proteins, and sub-spine structure, e.g., endosomes—at postsynaptic densities (Spence and Soderling, 2015; Bertling and Hotulainen, 2017; Dutta et al., 2021).

Structural changes occur within minutes from spine or dendritic shaft stimulation, including *ex novo* spine formation or spine enlargement. The first event is represented by calcium entrance inside the cell where it binds to Ca^2+^-binding protein calmodulin (CaM) which in turn activates the holoenzyme CaMKII (Lisman et al., 2012). Activated CaMKII forms complexes with postsynaptic density (PSD) molecules stabilizing NMDARs and enhancing AMPAR activity (Henley and Wilkinson, 2013) and expression (Opazo et al., 2010). At the same time, CaMKII interacts with key regulators of spine morphogenesis and LTP such as the small GTPase protein including Ras, RhoA, Rac1, and Cdc42 (Nishiyama and Yasuda, 2015). These GTPases activate several kinases including p21-activated kinase (PAK), Rho kinase (ROCK), and LIM kinase (LIMK), which in turn regulate actin remodeling and structural LTP *via* interaction and regulation of actin-binding partners such as Profilin, Cofilin, and Arp2/3 (Figure 1).

Long-lasting plasticity effects including spine enlargement and structural maintenance require *de novo* protein synthesis either *via* local protein synthesis (Holt et al., 2019; Runge et al., 2020) or *via* gene transcription in the nucleus through the activation of activity-dependent transcription factors such as CREB or MEF2C. Among neurotrophins, BDNF has a central role in numerous processes of functional and structural plasticity (Renna et al., 2022). BDNF *via* TrkB receptor binding and signaling regulates post-synaptic function by modifying NMDAR and AMPAR properties, hence favoring the induction and maintenance of LTP (Rauti et al., 2020) *via* actin cytoskeletal changes (Huang and Kandel, 2005; Rex et al., 2007). Indeed, BDNF–TrkB signaling has been shown to promote local protein synthesis of several proteins including Arc, Homer, LIMK1, which in end regulate the turnover of the dendritic actin cytoskeleton proteins (Ying et al., 2002; Messaoudi et al., 2007; Bramham, 2008).

Considering the aforementioned reviewed synaptic plasticity modifications induced by tDCS and also the activated molecular cascade underlying these effects, some recent works pointed the attention not only at the functional but also the structural changes (Paciello et al., 2018; Barbati et al., 2020; Gellner et al., 2020; Longo et al., 2022).

In this view, Paciello et al. (2018) have studied the effect of unilateral anodal tDCS of the auditory cortex (56 A/m^2^, 20 min, single daily sessions for 2 consecutive days) on healthy rats (normal-hearing, NH) and on rats with altered auditory cortex structural plasticity (i.e., animals exposed to acoustic trauma, NIHL). In this work comes up very clearly that tDCS exerts a global trophic action by increasing the number of dendritic spines in the auditory cortex, targeting apical dendrites of pyramidal neurons of layer II-III and V-VI in NH rats, while in NIHL rats, tDCS specifically targeted the most noise-affected layer II-III. In addition, the authors demonstrated that tDCS can modulate dendritic spine shape, increasing the number of both thin- and mushroom-shaped spines in NIHL, while in NH rats it increased only the number of thin spines. These results suggest that in the lesioned auditory cortex tDCS induces the formation of new spines but at the same time it stabilizes those already existing to preserve plasticity. TDCS-related structural changes were accompanied by increased synaptophysin levels and causally linked to increased level of BDNF in both NH and NIHL rats. TDCS effects on spinogenesis were, indeed, completely abolished in presence of ANA-12, the BDNF/TrkB receptor blocker.

Similarly, Barbati et al. (2020) showed increased spine density at both apical and basal dendrites of M1 layer II-III pyramidal neurons following repeated tDCS stimulation protocol (unilateral anodal tDCS 35.4 A/m^2^ for 20 min, single daily sessions for 3 consecutive days). This result was matched by enhanced forelimb strength and motor skilled performance and by increased synaptic transmission and plasticity at M1 layer II-III horizontal connections in tDCS-mice compared with the relative sham-controls. At the cellular level, tDCS increased neurotransmitter release and AMPA/NMDA ratio, events that are consistent with the increased spine density observed in tDCS mice. At the molecular level, the dendritic spine structural changes were accompanied by an increased level of BDNF and by an increased level of phosphorylation of key synaptic and structural plasticity players including CaMKII, CREB, and GluA1. Moreover, tDCS activated nitric oxide pathway leading to GluA1 S-nitrosylation and consecutively GluA1 phosphorylation at Ser831 (pGluA1^*Ser*831^) thus increasing single-channel conductance (Selvakumar et al., 2013; Barbati et al., 2020). As a proof-of-concept, the authors showed that mouse treatment with the NOS inhibitor, L-NAME, abolished tDCS-induced increases of pGluA1^*Ser*831^. This effect likely cooperated with other tDCS-induced epigenetic mechanisms including the pCREB-dependent recruitment of the histone acetyltransferase, CBP, at the promoter region of the *BDNF* gene (Figure 1).

These recent works provided the first evidence on how tDCS promotes not only functional but also structural plasticity changes at dendritic spines encouraging further investigation to clarify the molecular mechanisms at the basis of the actin remodeling events induced by tDCS and to focus on tDCS effects on brain connectivity.

## Transcranial direct current stimulation and rewiring

Recently, there has been growing interest in “brain connectome” to understand how brain and therefore neuronal structures gives rise to brain function, and ultimately, to behavior. The outline connection between neurons, determines how the stream of information flows through neural circuits, and therefore, how these circuits function (Sporns et al., 2005; Bota et al., 2015).

Recent works indicate that in the mammalian brain neuronal connections may undergo rewiring during learning and experience-dependent plasticity (Bennett et al., 2018). This network reorganization reinforces some neuron-to-neuron connections and weakened some others by synapse elimination.

Rewiring is a phenomenon that consists of two different mechanisms. The first one includes formation and elimination of individual synapses at existing connections determining a local event where the number of synapses at the connection change. The second type involves a radical reconfiguration of neural connection by incorporating or removing neurons from the existing circuit (Barnes and Finnerty, 2010). The functional and structural changes on the basis of this phenomenon are the consequence of electrical activity and hence, rewiring provides the strategy through which the brain responds to experience in a long-lasting way.

Given tDCS ability to modulate cortical excitability and to promote plasticity mechanisms by changing synaptic efficacy and structure some works have pointed attention to its ability to modulate brain connectivity.

In line with this, Polanía et al. (2011) showed that, on healthy subjects, unilateral anodal tDCS (0.625 A/m^2^, 10 min) has an effect on functional network synchronization not only within the target motor area but also coupling the former with neighbored premotor and sensorimotor areas.

Neuroplastic events and the reorganization of motor cortical connections have also been described as crucial processes in stroke recovery (Rossini et al., 2003; Murphy and Corbett, 2009; Xing and Bai, 2020). In patients with stroke, cortical reorganization, with an increased excitability of the contralesional hemisphere has been observed recurrently (Butefisch et al., 2003, 2008). Therefore, good functional recovery has frequently been associated with a rebalancing of interhemispheric inhibition (Nowak et al., 2009; Calabrò et al., 2019). As such, the most common tDCS configuration used in human and rodent stroke studies is the bilateral stimulation with the anode over the lesioned cortex and cathode over the contralateral side, providing the simultaneous stimulation of the two cortices with facilitating and inhibiting currents on affected and unaffected hemispheres, respectively.

In keeping with this, bilateral tDCS effects in stroke recovery have been tested in clinical studies showing encouraging results (Chew et al., 2020; Orrù et al., 2020). Furthermore, works investigating tDCS capacity to induce changes in cortical electroencephalogram oscillations, suggested that motor recovery might be enhanced by early stimulation that seeks to increase functional connectivity (FC) of motor relays and pathways (Bolognini et al., 2020; bilateral tDCS delivered at 0.57 A/m^2^, 15 min; Vecchio et al., 2018; bilateral tDCS delivered at 0.40 A/m^2^, 12 min). Lefebvre et al. (2017) showed that one single session of bilateral tDCS (0.28 A/m^2^, 30 min) applied over M1-modulated FC in patients with stroke. In particular, seed-based analysis of FC established that tDCS-enhanced FC within the motor and premotor regions in the lesioned hemispheres 1 week after the end of the stimulation protocol.

Moreover, in a recent preclinical study, Longo et al. (2022) applied bilateral tDCS over M1 (35.4 A/m^2^, 20 min, single daily sessions for 3 consecutive days) in a mouse model of ischemic stroke and showed that tDCS accelerated motor recovery by enhancing forelimb strength and ameliorating performances in both skilled and non-skilled motor tasks. The authors looked at possible effects on FC by recording local field potentials through epidurally implanted electrodes over M1 and somatosensory cortices and analyzing total coherence (TotCoh)—an index expressing global functional coupling of the LFP rhythms (Vecchio et al., 2019). Interestingly, they found that functional coupling between M1 and somatosensory cortices of both the hemispheres was decreased at all frequency bands and time points in stroke mice and, more importantly, tDCS significantly increased connectivity. Particularly, tDCS in stroke mice restored TotCoh values back to those observed in sham-healthy mice and, in addition, increased this parameter in healthy mice, suggesting that a structural network reorganization occurs following tDCS. In support of this, the analysis of Golgi-Cox staining of the peri-infarct cortex showed increased spine density at both apical and basal dendrites at layer II-III pyramidal neurons following tDCS, a result that corroborates the mounting evidence of tDCS- induced spinogenesis (Barbati et al., 2020; Gellner et al., 2020). Notably, the authors provided a causal link between BDNF and tDCS-dependent effects by demonstrating that blockade of BDNF/TrkB receptor hindered: (i) improvements of functional outcomes; (ii) increases in spine density and TotCoh, and also (iii) the activation of plasticity related proteins, such as ERK, CaMKII, and MEF2C.

These results provide the first evidence linking tDCS effects on structural plasticity to changes in connectivity, substantiating a novel role for tDCS in shaping neuronal connections. Given the translational relevance of such a tool, further studies are warranted to look more in-depth at tDCS effects on the FC by advanced analysis (e.g., graph theory) on EEG and functional MRI signals and at mechanism of action on dendritic spine structure and dynamics to optimize its use to modulate, preserve, and restructure neural circuits.

## Conclusion

In the last two decades, tDCS’ positive impact on the cognitive and motor functions has been proven in the subjects with diverse psychiatric and neurological disorders. This NIBS has also been used on animal models to clarify the mechanisms behind its effects on brain plasticity and to understand, more broadly, the relationship between structural and functional changes at synapses.

In literature, tDCS has been shown to modulate synaptic plasticity and, at the same time, to regulate the expression, activation, and localization of plasticity-related proteins such as BDNF, CaMKII, and GluA1. Intriguingly, these molecules are part of well-known signaling pathways at the basis of spine remodeling and actin polymerization.

Recent works have indeed shown that tDCS can modify the number and structure of dendritic spine, indicating that this NIBS not only changes the efficacy of a synapse, but it also shapes it. These structural changes at synapses might favor rewiring processes improving the neuronal networks and resulting in enhanced learning and memory as well as amelioration of cognitive and motor functions clinically and experimentally observed following tDCS.

In conclusion, in this review, we recapitulated the most recent evidence around tDCS’ plastic effects bringing into focus its capability to trigger dendritic spine rearrangement in neurons, thus, changing neuronal wiring patterns. Currently, treatments based on boosting rewiring are explored by the scientific community to treat acute brain damage as well as permanent and progressive brain dysfunctions. A deeper comprehension of tDCS effects on rewiring and structural plasticity at the basis of neuronal network rearrangement will allow tDCS to be exploited therapeutically for treating brain pathologies characterized by the widespread brain wiring abnormalities.

## Author contributions

SAB conceived the work and drafted the manuscript. MVP conceived the work and contributed making a critical revision of the manuscript. CG contributed by making a critical revision of the manuscript. All authors read and approved the final version of the review.
